# Genome resequencing and comparative variome analysis in a *Brassica rapa* and *Brassica oleracea* collection

**DOI:** 10.1038/sdata.2016.119

**Published:** 2016-12-20

**Authors:** Feng Cheng, Jian Wu, Chengcheng Cai, Lixia Fu, Jianli Liang, Theo Borm, Mu Zhuang, Yangyong Zhang, Fenglan Zhang, Guusje Bonnema, Xiaowu Wang

**Affiliations:** 1 Institute of Vegetables and Flowers, Chinese Academy of Agricultural Sciences, Key Laboratory of Biology and Genetic Improvement of Horticultural Crops of the Ministry of Agriculture, Sino-Dutch Joint Laboratory of Horticultural Genomics, Beijing 10081, China; 2 Wageningen UR Plant Breeding, Wageningen University and Research centre, Droevendaalsesteeg 1, Wageningen 6708 PB, The Netherlands; 3 Beijing Academy of Agriculture and Foresty Science (BAAFS), Beijing Vegetable Research Center (BVRC), Beijing 10089, China

**Keywords:** Plant domestication, Agricultural genetics

## Abstract

The closely related species *Brassica rapa* and *B. oleracea* encompass a wide range of vegetable, fodder and oil crops. The release of their reference genomes has facilitated resequencing collections of *B. rapa* and *B. oleracea* aiming to build their variome datasets. These data can be used to investigate the evolutionary relationships between and within the different species and the domestication of the crops, hereafter named morphotypes. These data can also be used in genetic studies aiming at the identification of genes that influence agronomic traits. We selected and resequenced 199 *B. rapa* and 119 *B. oleracea* accessions representing 12 and nine morphotypes, respectively. Based on these resequencing data, we obtained 2,249,473 and 3,852,169 high quality SNPs (single-nucleotide polymorphisms), as well as 303,617 and 417,004 InDels for the *B. rapa* and *B. oleracea* populations, respectively. The variome datasets of *B. rapa* and *B. oleracea* represent valuable resources to researchers working on evolution, domestication or breeding of *Brassica* vegetable crops.

## Background & Summary

Many important crops have been domesticated and are cultivated from the genus *Brassica*, including those used as oilseeds, condiments, fodder, or vegetables. The ‘triangle of U’^1^ describes the relationships between the six economically important *Brassica* species. Three of the six species are diploids (A genome, *Brassica rapa*, *n*=10; B genome, *B. nigra*, *n*=8; and C genome, *B. oleracea*, *n*=9), while the other three species are allotetraploids resulting from interspecific hybridization between the diploids (AB genomes, *B. juncea*, *n*=18; AC genomes, *B. napus*, *n*=19; BC genomes, *B. carinata*, *n*=17). These different crops are characterized by their specialized development of organs^2^, and are referred to as morphotypes. Interestingly, similar morphotypes are often selected for in two or more *Brassica* species, clearly illustrating convergent crop domestication. This includes the leafy heads in Chinese cabbage (*B. rapa*) and cabbage (*B. oleracea*) and tubers at stems or hypocotyls/roots in kohlrabi’s (*B. oleracea*), swede’s (*B. napus*) and turnips (B*. rapa*), enlarged stems in marrow stem kale (*B. oleracea*) and also broccoletto (*B. rapa*), enlarged inflorescences in cauliflower and broccoli’s (*B. oleracea*), the many axillary shoots in Brussels sprouts (*B. oleracea)* and mizuna/mibuna’s (*B. rapa*) and the increased numbers of enlarged seedpods in oilseed (*B. rapa* and *B. napus*).

In recent years, the genomes of *B. rapa* and *B. oleracea* have been assembled and released^3–5^. Brassicaceae comparative genomics provided 24 genomic blocks (GBs) as the framework for genome studies in *Brassica*s^6^. With the GBs system, the comparative genomic analysis of *B. rapa*, *B. oleracea*, and *A. thaliana* revealed the whole genome triplication (WGT) event that is shared by all *Brassica*s^7–9^, and made it possible to deduce and reconstruct the diploid ancestor for the Brassiceae tribe^10^. Further studies reported the phenomenon of sub-genome dominance among the three sub-genomes in *Brassica*s^8,9^, and found that the biased distribution of small RNA-targeted TEs plays an important role in this phenomenon^11,12^. Furthermore, the release of the genome sequences has also contributed to the mapping, cloning and functional studies of agronomic genes in *Brassica* crops, such as two clubroot resistance genes^13,14^, two blackleg resistance genes^15,16^, as well as two key genes regulating the time of flowering^17–19^. The availability of these reference genomes also made it possible to build variome datasets by resequencing populations of *B. rapa* and *B. oleracea*, to investigate domestication of the diverse morphotypes.

**Figure 1:**
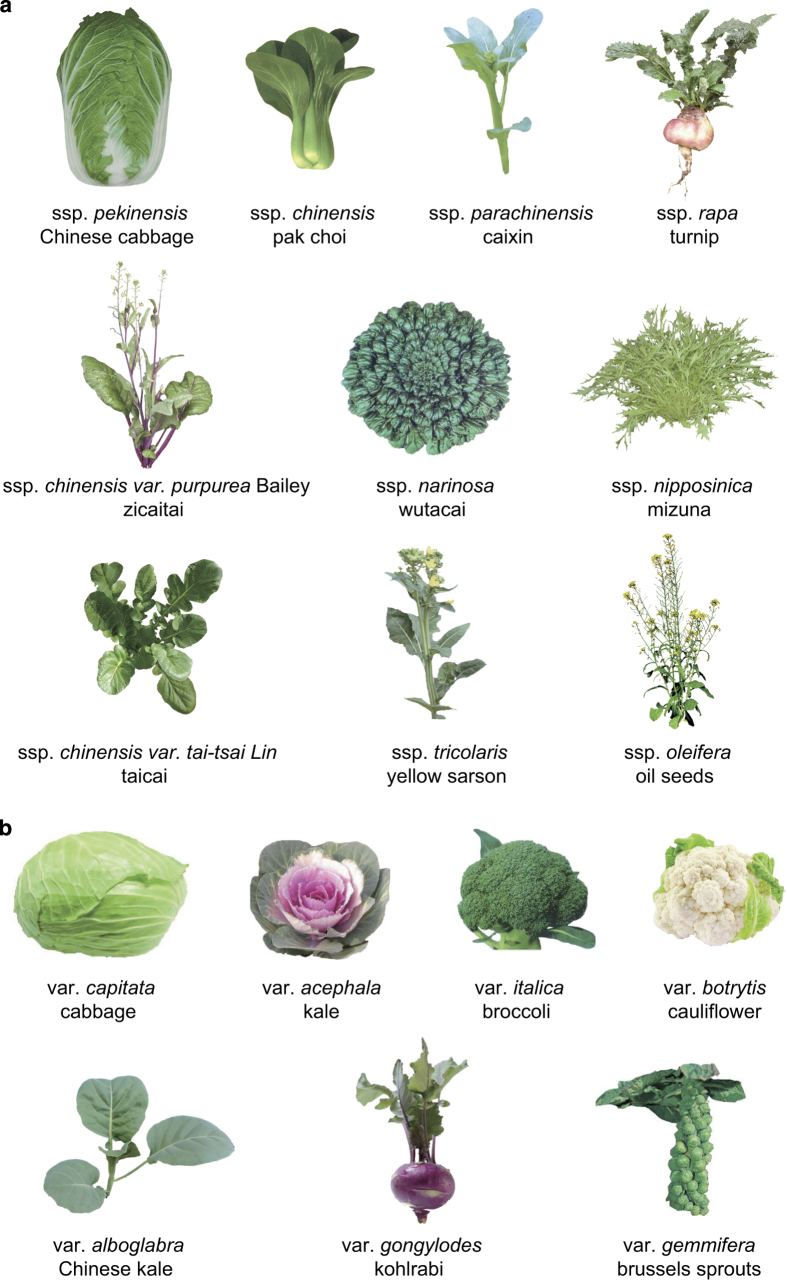
Images of the different morphotypes of *Brassica* vegetables. (**a**) ten images show representative morphotypes of *B. rapa*; (**b**) seven images show representative morphotypes of *B. oleracea*.

Here, we selected 199 *B. rapa* and 119 *B. oleracea* accessions representing the 12 and nine morphotypes from these two species, respectively (Fig. 1, Supplementary Tables 1 and 2). These accessions originate from a wide geographic range, from North till South Asia and Europe^20^. Whole genome resequencing data of these 199 *B. rapa* and 119 *B.oleracea* accessions was generated using the Illumina HiSeq2000 platform, with paired-end reads from 350 bp insert libraries. After filtering low-quality or duplicated reads, totally, we produced 982.87 Gb resequencing data for the 199 *B. rapa* accessions and 742.35 Gb for the 119 *B. oleracea* accessions, which is about on average 10× coverage for accessions of both *B. rapa* and *B. oleracea*, ranging from 1.67× till 19.8× coverage. These reads were then mapped to the reference genomes of *B. rapa* version V1.5 and *B. oleracae* V1.0, respectively. Finally, we called out 2,249,473 and 3,852,169 reliable SNPs (single-nucleotide polymorphisms), as well as 303,617 and 417,004 InDels for the *B. rapa* and *B. oleracea* populations, respectively. With these datasets, we have identified genomic selection signals for traits of leaf-heading and tuberous organs in both *B. rapa* and *B. oleracea*, and found that sub-genome parallel selection was associated with morphotype diversification and convergent crop domestication in the two *Brassica* species^21^.

The whole genome resequencing data and variome datasets for the *B. rapa* and *B. oleracea* populations are valuable resources to researchers studying evolution, domestication or trait discovery, and to breeders of *Brassica* crops. These data can be exploited to develop genetic markers, and chip arrays for gene mapping and functional studies.

## Methods

These methods are expanded versions of descriptions in our related work^21^.

### Sample collection of *B. rapa* and *B. oleracea*

Accessions representing the 12 morphotypes of *B. rapa* and nine morphotypes of *B. oleracea* were collected (Tables 1 and 2). Almost all morphotypes from the two *Brassica* species were included in this collection (Supplementary Tables 1 and 2). The 199 *B. rapa* accessions included 46 Chinese cabbage accessions (*B. rapa* ssp. *pekinensis*) (Table 1), 54 turnip accessions (*B. rapa* ssp. *rapa*), 25 pak choi accessions (*B. rapa* ssp. *chinensis*), 30 caixin accessions (*B. rapa* ssp. *parachinensis*), 13 zicaitai accessions (*B. rapa* ssp. *chinensis var. purpurea* Bailey), 14 oil seed accessions (*B. rapa* ssp. *oleifera*), four taicai accessions (*B. rapa* ssp. *chinensis var. tai-tsai Lin*), seven wutacai accessions (*B. rapa* ssp. *narinosa*), one edible flower accession (*B. rapa* ssp. *broccoletto*) and one yellow sarson (*B. rapa* ssp. *tricolaris*), as well as two accessions each of komatsuna (*B. rapa* ssp. *perviridis*) and mizuna (*B. rapa* ssp. *nipposinica*). This *B. rapa* population includes 75 DH (doubled haploid) lines, 18 inbred lines, and 106 germplasm lines (Supplementary Table 1). The 119 *B. oleracea* accesions included 45 cabbage accessions (*B. oleracea* var. *capitata*) (Table 2), 19 kohlrabi accessions (*B. oleracea* var. *gongylodes*), 20 cauliflower accessions (*B. oleracea* var. *botrytis*), 23 broccoli accessions (*B. oleracea* var. *italica*), four Chinese kale accessions (*B. oleracea* var. *alboglabra*), as well as two accessions each of kale (*B. oleracea* var. *acephala*), Brussels sprouts (*B. oleracea* var. *gemmifera*), curly kale (*B. oleracea* var. *sabellica*), and wild *B. oleracea*. This *B. oleracea* population included 69 DH or inbred lines, 44 germplasm lines (inbred lines from genebank accessions), and six genebank accessions (Supplementary Table 2).

We have grown all the 199 *B. rapa* accessions with three replicates in the green house during autumn 2014 to confirm their morphotypes. The phenotypes of these accessions were investigated till their maturity.

### Sample preparation and resequencing

Plants of the 199 *B. rapa* accessions were grown in a greenhouse, each accession with five replicates. At the six-leaf stage, the two youngest leaves were collected from one of the five plants and DNA was extracted from these leaves. For *B. oleracea*, the DNA of the 44 germplasm lines and six genebank accessions was extracted as described for *B. rapa*. However for the DH and inbred lines, 50–100 seedlings per genotype were grown on moist filter paper. Cotyledons and hypocotyls were harvested after 12 days and DNA was then extracted.

DNA libraries with approximately 350 bp insert sizes were constructed following the manufacturer’s instructions (Illumina GAII) and paired-end resequencing reads were generated by commercial Illumina HiSeq 2000 service provided by Biomarker Technologies Corporation, Beijing, and BGI, Shenzhen.

### Raw data were filtered before alignment

We filtered the raw reads before alignment to the corresponding reference genomes of *B. rapa*^3^ or *B. oleracea*^5^. First, low quality reads were filtered based on the following three rules. If one end of a paired-end read had >5% ‘N’ bases, then the paired-end read was removed. Secondly, for each paired-end read pair, if one of two reads had an average base-quality less than 20 (Phred-like score), then both reads were removed. Thirdly, for each read, we trimmed its 3’ bases if the quality scores are below 13. The trimming was stopped at the base with quality score ≥13. After trimming, if the remaining read was less than 40, the paired-end reads were removed. Moreover, considering that the duplicates of paired-end reads were generated from a single amplicon, we further removed redundant duplicated reads and only kept a single pair. We completed this raw read filtering process using an in-house made Perl script.

### Alignment and variants calling

Filtered reads of each re-sequenced sample were mapped onto the corresponding reference genomes using the software Burrows-Wheeler Aligner (BWA version 0.7.5a-r405)^22^. We used the *B. rapa* genome version 1.5 and *B. oleracae* V1.0 as the references for the two species^4^. The reference genomes were first indexed by using commands as ‘bwa index reference.fa’ and ‘samtools faidx reference.fa’. The clean reads of each accession were then mapped to the indexed reference genomes one by one with the algorithm ‘mem’ of BWA. The command line was ‘bwa mem reference.fa sample_1.fq sample_2.fq>sample.sam’. It generated a sam file ‘sample.sam’ as the mapping output for each accession. This sam file can be handled by Samtools^23^ to call variants.

Samtools was used to call SNPs and InDels for each resequenced *Brassica* accession from the mapping results reported by BWA.

Samtools (version 0.1.19–44428 cd)^23^ was first used to transfer the sam file to bam file by command ‘samtools view -bS sample.sam sample.bam’. The bam file was then sorted by ‘samtools sort sample.bam sample.sorted.bam’. After index the sorted bam file with ‘samtools index sample.sorted.bam’, candidate genomic variants were called out by using the algorithm ‘mpileup’ of Samtools. The full command was ‘samtools mpileup -q 20 -Q 30 -ugf reference.index sample.sorted.bam | bcftools view -p 0.9 -cg ->candidateVariants.list’. We set ‘-q 20’ to use nucleotides with Phred-like quality scores higher than 20 as reliable nucleotides of a read to report variants. ‘-Q 30’ denotes that mapping quality of reads should be higher than 30 to be considered as a reliable mapping. Bcftools was used to transfer the vcf file generated by ‘mpileup’ and reported candidate variants in an output file^23^. We used ‘-p 0.9’ to ask Bcftools to report variants at a locus with more than 10% reads showing a different genotype to that of the reference.

**Figure 2:**
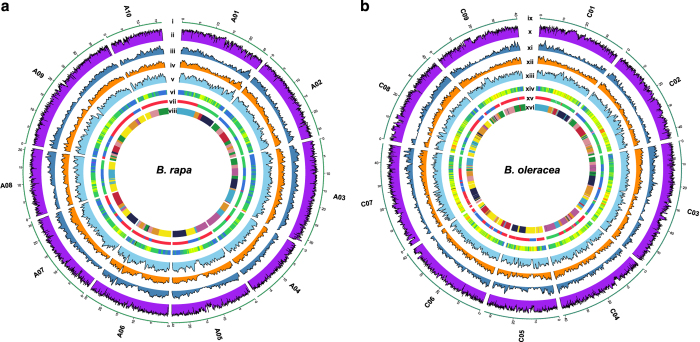
The distribution of genomic variations on chromosomes of the resequenced populations of 199 *B. rapa* and 119 *B. oleracea* accessions. (**a**) The genomic information of the *B. rapa* population; ***i***: the ten chromosomes of *B. rapa*, the physical positions are indicated in units of million bases; ***ii***: the genomic heterozygosity of the *B. rapa* population. Area charts quantify ***iii***: functional SNPs/InDels, ***iv***: InDels, ***v***: SNPs. ***vi***: heat map for gene density. ***vii***: subgenome partition of the *B. rapa* genome, red, green, and blue corresponding to subgenomes LF, MF1, and MF2, respectively^10^. ***viii***: The triplicated 24 genomic blocks in *B. rapa*^10^. (**b**) The genomic information of the *B. oleracea* population; ***ix***: the nine chromosomes of *B. oleracea*; ***x***: the genomic heterozygosity of the *B. oleracea* population. Area charts quantify ***xi***: functional SNPs/InDels, ***xii***: InDels, ***xiii***: SNPs. ***xiv***: heat map for gene density. ***xv***: subgenome partition in *B. oleracea* genome, red, green, and blue corresponding to subgenomes LF, MF1, and MF2, respectively^5^. ***xvi***: The triplicated 24 genomic blocks in *B. oleracea*^5^. LF denotes the least fractionated subgenome, MF1 and MF2 denote for more fractionated sub-genomes 1 and 2 (ref. 3).

We further filtered the candidate variant list for reliable variants. For each accession, we screened its variations one by one. Here, we called the genotype that is the same as the reference ‘reference allele’, while the one that is different to the reference as ‘alternative allele’. For each variant, only alleles that were covered by sufficient reads (≥3 reads) were considered as confident alleles, and the variant was kept as a reliable variant. We performed this process for all *Brassica* accessions. This removed potentially false variants produced by sequencing errors. Since most of the *Brassica* samples (except six genebank accessions of *B. oleracea*) are from homozygous or almost homozygous accessions, we filtered our data further by retaining the variant calls that were homozygous in individual homozygous accessions (loci were considered as heterozygous if 0.2>D_R_/(D_A_+D_R_)>0.8, where D_R_ denotes the number of reads with the reference allele, and D_A_ denotes the number of reads with the alternative allele). We developed in-house Perl scripts to complete the candidate variants filtering processes. In order to remove rare SNPs and use common SNPs to scan for selection signals, we filtered out SNPs that had a MAF (minor allele frequency)<0.05 in both *B. rapa* and *B. oleracea*. Totally, we obtained 2,249,473 SNPs and 303,617 InDels for the *B. rapa* population, as well as 3,852,169 SNPs and 417,004 InDels for the *B. oleracea* population. For the two accessions with lowest re-sequencing depth in *B. rapa* and *B. oleracea*, we still obtained genotypes of 1,577,624 and 2,690,136 SNPs, respectively. We plotted genomic heterozygosity, density of SNPs and InDels along with the gene density to show the distribution features of these variants in the two *Brassica* genomes (Fig. 2).

### Functional annotation of genomic variants

**Figure 3:**
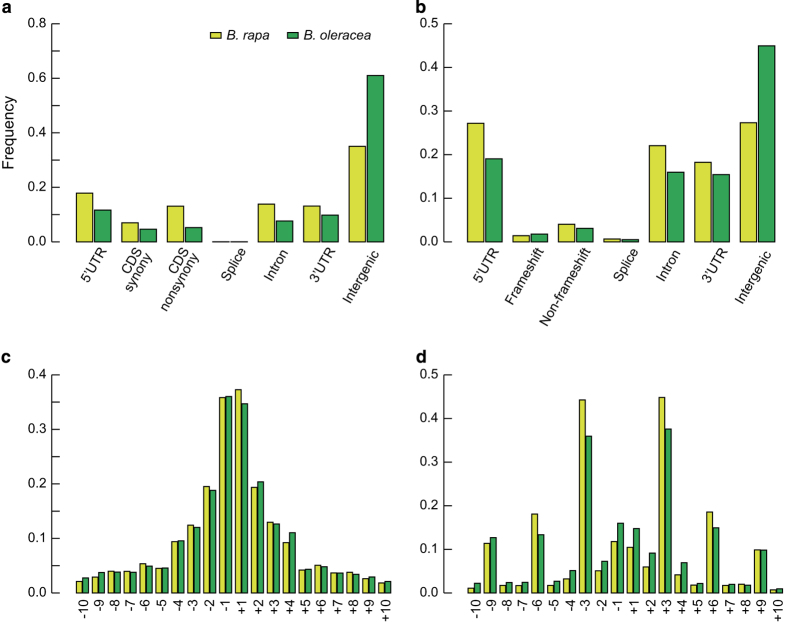
The distribution of genomic variations (SNPs and InDels) determined from the resequenced accessions of *B. rapa* and *B. oleracea.* The distribution of 2,148,937 and 3,471,818 SNPs (**a**), as well as 312,618 and 276,587 InDels (**b**) in genomes of *B. rapa* and *B. oleracea*, respectively. The length distributions of total InDels (**c**), as well as 17,018 and 13,502 InDels located at coding regions (**d**) in genomes of *B. rapa* and *B. oleracea*, respectively. On the X-axis of (**c**,**d**), ‘−’ denotes deletion variants in numbers of bases, while ‘+’ denotes insertion variants in numbers of bases.

The variants identified in the two *Brassica* genomes were further annotated into different groups (Table 3 and Table 4). According to the genomic positions of SNPs and InDels relevant to predicted gene models, we first separated them into 1) variants located at genic regions and 2) at inter-genic regions. Variants located at genic regions were further separated into three subgroups: a) variants in coding sequences (CDs), b) in introns and c) in untranslated regions (UTRs). The SNPs and InDels located at CDs (1a) were classified into two subgroups: the subgroup causing changes to the coding amino acids, including non-synonymous SNPs and frame shift InDels, and the subgroup causing no changes to the amino acids, including synonymous SNPs and InDels that do not cause frame-shifts. Intronic variants (1b) were also separated into two subgroups: causing (8 bp to the splice site) or not causing splice site mutations; UTR variants (1c) are divided into two subgroups: variants in 5′ or 3′UTR regions (Fig. 3a,b). The results show that among these 2,249,473 SNPs and 303,617 InDels in *B. rapa*, 161,319 SNPs (nonsynonymous and splice site) and 16,905 InDels (CDS and splice site) respectively, introduced changes to the protein sequences (Table 3); for the 3,852,169 SNPs and 417,004 InDels in *B. oleracea*, 154,863 SNPs and 16,687 InDels respectively introduced changes to the protein sequences (Table 4). Additionally, we analyzed the length distribution of InDels, and found that 1 bp deletions or insertions are the dominant InDels (Fig. 3c) in both *Brassica* populations. The length distribution of InDels located in coding regions of genes was also investigated, and it was found that InDels of three or fold changes of three nucleotides are clearly the dominant types (Fig. 3d). These InDels will not introduce frame-shift mutations to genes, thus are under less stringent selection compared to InDels that don’t correspond to fold changes of three.

We further investigated the genetic diversity within each morphotype group in both species with the annotated genomic variations. We performed the analysis by counting the number of polymorphic variants in each morphotype group. The results showed that groups of turnip (54) and Chinese cabbages (46) in *B. rapa* had most polymorphic loci based on both total SNPs as well as functional SNPs (non-synonymous SNPs or SNPs located at splicing sites) (Supplementary Table 3). Interestingly, the groups of turnip and pak choi had the most polymorphic loci based on total InDels and functional InDels (InDels located at coding sequences or splicing sites). For *B. oleracea*, groups of cabbage (45) and kohlrabi (19) had both most polymorphic loci of SNPs and InDels (Supplementary Table 4). The number of polymorphic variants reflects the genetic diversity in each group. However, the number of variants is also impacted by the numbers of samples studied.

### Code availability

Custom perl scripts used to filter raw reads and candidate genomic variants can be downloaded through http://brassicadb.org/brad/datasets/pub/ReseqPars/codes/ in *Brassica* database BRAD, or are freely available when requested by email to the authors. Other tools used in this work including version details are BWA (version 0.7.5a-r405) and SAMtools (version 0.1.19–44428 cd). Command lines with details of parameters when using these tools are described in the method section.

## Data Records

All the raw reads of 199 *B. rapa* and 119 *B. oleracea* accessions used in this work have been deposited in the NCBI Sequence Read Archive with the accession ID SRP071086 (Data Citation 1). The SNP and InDel datasets identified on the resequencing data and related vcf files are public available in *Brassica* Database BRAD (Data Citation 2).

## Technical Validation

In order to measure the quality of variants called out from the resequencing data, we selected five SNPs (Table 5), and genotyped 95 out of the 199 *B. rapa* accessions for these SNPs using the method of KASP (kompetitive allele specific PCR). With the same method, we also test the polymorphic level of five SNPs determined from 119 *B. oleracea* accessions in another group of 281 *B. oleracea* accessions. KASP is a homogeneous, fluorescence-based endpoint SNP genotyping platform (LGC Genomics LLC, Beverly, MA, USA). The five selected SNPs satisfied the following criteria of KASP experiments: 1) No other genomic matches were found for the 50 bp sequences flanking the two sides of each candidate SNP; 2) There are no other SNPs or InDels located at the 50 bp flanking regions of the candidate SNP.

For each SNP, two allele-specific primers A1 and A2 and one common reverse primer C were designed by LGC Genomics. The PCR reaction mixture had a total volume of 5 μl and included 2.5 μl DNA, 2.5 μl 2×master mix with 0.07 μl primer mix according to the manufacturer’s guidelines. Three no-template controls were included for each SNP locus. The amplification process was ran in Gene Amp PCR System 9700 (Applied Biosystems) using the following program: 94 °C for 15 min followed by 10 touchdown cycles of 94 °C for 20 s, 61–55 °C for 60 s (decreasing by 0.6 °C per cycle), followed by 26 cycles of 94 °C for 20 s, 55 °C for 60 s. Fluorescence detection was then performed in a 7900 HT Fast Real-Time PCR System (Applied Biosystems), and the results were analysed using SDS2.3 Software (supplied by Applied Biosystems).

We further compared the genotypes of the five SNPs in the 95 samples that were called out by resequencing data, with the genotypes of these loci that were reported by the KASP experiments. Results show that all the 475 loci analyzed have consistent genotypes between the two methods of resequencing and the KASP experiment (Table 5), supporting the fact that the genomic variants are of high quality. The polymorphism level of the five SNPs in *B. oleracea* is shown in Supplementary Table 5.

## Usage Notes

All the resequencing datasets of this work are deposited in the NCBI database with the accession number SRP066057 (alias: PRJNA301642). Access to the plant materials (or DNA samples for materials provided from companies) described in this paper is possible for research purposes via a material transfer agreement with the material owners. Please contact Dr Xiaowu Wang (wangxiaowu@caas.cn) or Guusje Bonnema (guusje.bonnema@wur.nl).

## Additional Information

**How to cite this article:** Cheng, F. *et al.* Genome resequencing and comparative variome analysis in a *Brassica rapa* and *Brassica oleracea* collection. *Sci. Data* 3:160119 doi: 10.1038/sdata.2016.119 (2016).

**Publisher’s note:** Springer Nature remains neutral with regard to jurisdictional claims in published maps and institutional affiliations.

## Supplementary Material



Supplementary Tables

## Figures and Tables

**Table 1:** Summary of the number of accessions per *B. rapa* morphotype.

**Index**	**Name**	**English name morphotype**	**Number**
1	ssp. *pekinensis*	Chinese cabbages	46
2	ssp. *rapa*	turnip	54
3	ssp. *chinensis*	pak choi	25
4	ssp. *parachinensis*	caixin	30
5	ssp. *chinensis var. purpurea* Bailey	zicaitai	13
6	ssp. *oleifera*	oil seeds	14
7	ssp. *chinensis var. tai-tsai Lin*	taicai	4
8	ssp. *narinosa*	wutacai	7
9	ssp. *broccoletto*	edible flower	1
10	ssp. *tricolaris*	yellow sarson	1
11	ssp. *perviridis*	komatsuna	2
12	ssp. *nipposinica*	mizuna	2
Total	/	/	199

**Table 2:** Overview of the numbers of accessions per *B. oleracea* morphotype.

**Index**	**Name**	**English name morphotype**	**Number**
1	var. *capitata*	cabbage	45
2	var. *gongylodes*	kohlrabi	19
3	var. *botrytis*	cauliflower	20
4	var. *italica*	broccoli	23
5	var. *alboglabra*	Chinese kale	4
6	var. *acephala*	kale	2
7	var. *gemmifera*	brussels sprouts	2
8	var. *sabellica*	curly kale	2
9	wild type	wild	2
Total	/	/	119

**Table 3:** The distribution of SNPs in the 199 *B. rapa* and 119 *B. oleracea* accessions.

	**Genic**	**Intergenic**	**Total**					
	**CDS**		**Intron**		**UTR**			
	**nonSyn**	**Syn**	**splice**	**intron**	**5UTR**	**3UTR**		
*B. rapa*	160,639	298,671	680	320,898	402,207	297,313	769,065	2,249,473
*B. oleracea*	154,038	176,338	825	270,474	474,953	373,303	2,402,238	3,852,169
Abbreviations: CDS for coding sequences; nonSyn for non-synonymous; Syn for synonymous; UTR for untranslated region.								

**Table 4:** The distribution of InDels in the 199 *B. rapa* and 119 *B. oleracea* accessions.

	**Genic**	**Intergenic**	**Total**					
	**CDS**		**Intron**		**UTR**			
	**Frameshift**	**Nonframeshift**	**splice**	**intron**	**5UTR**	**3UTR**		
*B. rapa*	4,467	10,728	1,710	68,514	81,779	56,341	82,441	303,617
*B. oleracea*	5,724	9,516	1,447	57,427	82,447	59,386	203,441	417,004
Note: frameshift means an InDel variation that causes a frameshift mutation; abbreviations are the same as in Table 3.								

**Table 5:** Genotype verification of five SNP loci in 95 *B. rapa* accessions.

**Loci**	**Chromosome**	**Position**	**#Samples**	**Ratio of correction**
1	A01	8278402	95	100%
2	A05	5655627	95	100%
3	A08	8149675	95	100%
4	A08	8028973	95	100%
5	A10	1610572	95	100%
Total	/	/	475	100%
