# Supplementary material for: Genome resequencing and comparative variome analysis in a Brassica rapa and Brassica oleracea collection
Source: Sci Data. 2016 Dec 20;3:160119. doi: 10.1038/sdata.2016.119 (PMC5170593; doi:10.1038/sdata.2016.119)
Supplement: Supplementary Tables [file sdata2016119-s2.doc]

**Supplementary Table 1**. The resequencing and material information of 199 *B. rapa* accessions.

| **Index** | **Name** | **English name**  **morphotype** | **Type *** | **Resequencing**  **data (Gb)** |
| --- | --- | --- | --- | --- |
| 1 | ssp. *pekinensis* | Chinese cabbage | DH line | 1.81 |
| 2 | ssp. *pekinensis* | Chinese cabbage | DH line | 2.11 |
| 3 | ssp. *pekinensis* | Chinese cabbage | DH line | 2.4 |
| 4 | ssp. *pekinensis* | Chinese cabbage | Inbred line | 2.28 |
| 5 | ssp. *pekinensis* | Chinese cabbage | DH line | 2.24 |
| 6 | ssp. *pekinensis* | Chinese cabbage | DH line | 2.75 |
| 7 | ssp. *pekinensis* | Chinese cabbage | DH line | 1.94 |
| 8 | ssp. *pekinensis* | Chinese cabbage | DH line | 2.61 |
| 9 | ssp. *pekinensis* | Chinese cabbage | Inbred line | 2.68 |
| 10 | ssp. *pekinensis* | Chinese cabbage | DH line | 2.38 |
| 11 | ssp. *pekinensis* | Chinese cabbage | Inbred line | 1.62 |
| 12 | ssp. *pekinensis* | Chinese cabbage | DH line | 2.72 |
| 13 | ssp. *pekinensis* | Chinese cabbage | DH line | 2.91 |
| 14 | ssp. *pekinensis* | Chinese cabbage | DH line | 2.12 |
| 15 | ssp. *pekinensis* | Chinese cabbage | DH line | 2.44 |
| 16 | ssp. *pekinensis* | Chinese cabbage | DH line | 2.57 |
| 17 | ssp. *pekinensis* | Chinese cabbage | DH line | 2.36 |
| 18 | ssp. *pekinensis* | Chinese cabbage | DH line | 2.26 |
| 19 | ssp. *pekinensis* | Chinese cabbage | DH line | 2.56 |
| 20 | ssp. *pekinensis* | Chinese cabbage | DH line | 2.34 |
| 21 | ssp. *pekinensis* | Chinese cabbage | Inbred line | 1.83 |
| 22 | ssp. *pekinensis* | Chinese cabbage | DH line | 2.75 |
| 23 | ssp. *pekinensis* | Chinese cabbage | DH line | 2.73 |
| 24 | ssp. *pekinensis* | Chinese cabbage | DH line | 2.32 |
| 25 | ssp. *pekinensis* | Chinese cabbage | Inbred line | 1.69 |
| 26 | ssp. *pekinensis* | Chinese cabbage | Inbred line | 2.3 |
| 27 | ssp. *pekinensis* | Chinese cabbage | DH line | 2.5 |
| 28 | ssp. *pekinensis* | Chinese cabbage | DH line | 2.56 |
| 29 | ssp. *pekinensis* | Chinese cabbage | DH line | 2.2 |
| 30 | ssp. *pekinensis* | Chinese cabbage | DH line | 2.46 |
| 31 | ssp. *pekinensis* | Chinese cabbage | DH line | 2.18 |
| 32 | ssp. *pekinensis* | Chinese cabbage | DH line | 3.31 |
| 33 | ssp. *pekinensis* | Chinese cabbage | DH line | 2.62 |
| 34 | ssp. *pekinensis* | Chinese cabbage | Inbred line | 1.42 |
| 35 | ssp. *pekinensis* | Chinese cabbage | DH line | 2.64 |
| 36 | ssp. *pekinensis* | Chinese cabbage | DH line | 2.96 |
| 37 | ssp. *pekinensis* | Chinese cabbage | DH line | 2.55 |
| 38 | ssp. *pekinensis* | Chinese cabbage | DH line | 2.59 |
| 39 | ssp. *pekinensis* | Chinese cabbage | DH line | 2.27 |
| 40 | ssp. *pekinensis* | Chinese cabbage | Inbred line | 2.19 |
| 41 | ssp. *pekinensis* | Chinese cabbage | DH line | 2.66 |
| 42 | ssp. *pekinensis* | Chinese cabbage | DH line | 2.47 |
| 43 | ssp. *pekinensis* | Chinese cabbage | DH line | 5 |
| 44 | ssp. *pekinensis* | Chinese cabbage | Inbred line | 2.46 |
| 45 | ssp. *pekinensis* | Chinese cabbage | Inbred line | 1.44 |
| 46 | ssp. *pekinensis* | Chinese cabbage | Germplasm | 3.87 |
| 47 | ssp. *rapa* | Turnip | DH line | 2.31 |
| 48 | ssp. *rapa* | Turnip | Inbred line | 1.42 |
| 49 | ssp. *rapa* | Turnip | DH line | 4.23 |
| 50 | ssp. *rapa* | Turnip | DH line | 10.82 |
| 51 | ssp. *rapa* | Turnip | DH line | 3.67 |
| 52 | ssp. *rapa* | Turnip | DH line | 3.8 |
| 53 | ssp. *rapa* | Turnip | Germplasm | 11.72 |
| 54 | ssp. *rapa* | Turnip | Germplasm | 13.71 |
| 55 | ssp. *rapa* | Turnip | Germplasm | 5.6 |
| 56 | ssp. *rapa* | Turnip | Germplasm | 8.53 |
| 57 | ssp. *rapa* | Turnip | Germplasm | 14.82 |
| 58 | ssp. *rapa* | Turnip | Germplasm | 8.61 |
| 59 | ssp. *rapa* | Turnip | Germplasm | 5.36 |
| 60 | ssp. *rapa* | Turnip | Germplasm | 16.76 |
| 61 | ssp. *rapa* | Turnip | Germplasm | 5.92 |
| 62 | ssp. *rapa* | Turnip | Germplasm | 5.34 |
| 63 | ssp. *rapa* | Turnip | Germplasm | 3.71 |
| 64 | ssp. *rapa* | Turnip | Germplasm | 7.73 |
| 65 | ssp. *rapa* | Turnip | Germplasm | 4.39 |
| 66 | ssp. *rapa* | Turnip | Germplasm | 6.23 |
| 67 | ssp. *rapa* | Turnip | Germplasm | 9.71 |
| 68 | ssp. *rapa* | Turnip | Germplasm | 3.4 |
| 69 | ssp. *rapa* | Turnip | Germplasm | 3.51 |
| 70 | ssp. *rapa* | Turnip | Germplasm | 2.55 |
| 71 | ssp. *rapa* | Turnip | Germplasm | 5.3 |
| 72 | ssp. *rapa* | Turnip | Germplasm | 7.53 |
| 73 | ssp. *rapa* | Turnip | Germplasm | 13.87 |
| 74 | ssp. *rapa* | Turnip | Germplasm | 12.72 |
| 75 | ssp. *rapa* | Turnip | Germplasm | 4.21 |
| 76 | ssp. *rapa* | Turnip | Germplasm | 4.53 |
| 77 | ssp. *rapa* | Turnip | Germplasm | 8.56 |
| 78 | ssp. *rapa* | Turnip | Germplasm | 6.74 |
| 79 | ssp. *rapa* | Turnip | Germplasm | 4.62 |
| 80 | ssp. *rapa* | Turnip | Germplasm | 10.8 |
| 81 | ssp. *rapa* | Turnip | Germplasm | 9.16 |
| 82 | ssp. *rapa* | Turnip | Germplasm | 5.07 |
| 83 | ssp. *rapa* | Turnip | Germplasm | 3.83 |
| 84 | ssp. *rapa* | Turnip | Germplasm | 5.21 |
| 85 | ssp. *rapa* | Turnip | Germplasm | 3.38 |
| 86 | ssp. *rapa* | Turnip | Germplasm | 4.06 |
| 87 | ssp. *rapa* | Turnip | Germplasm | 7.32 |
| 88 | ssp. *rapa* | Turnip | Germplasm | 2.27 |
| 89 | ssp. *rapa* | Turnip | Germplasm | 4.74 |
| 90 | ssp. *rapa* | Turnip | Germplasm | 2.52 |
| 91 | ssp. *rapa* | Turnip | Germplasm | 1.67 |
| 92 | ssp. *rapa* | Turnip | Germplasm | 2.64 |
| 93 | ssp. *rapa* | Turnip | Germplasm | 6.09 |
| 94 | ssp. *rapa* | Turnip | Germplasm | 4.34 |
| 95 | ssp. *rapa* | Turnip | Germplasm | 3.3 |
| 96 | ssp. *rapa* | Turnip | Germplasm | 5.22 |
| 97 | ssp. *rapa* | Turnip | Germplasm | 11.04 |
| 98 | ssp. *rapa* | Turnip | Germplasm | 7.61 |
| 99 | ssp. *rapa* | Turnip | Germplasm | 17.1 |
| 100 | ssp. *rapa* | Turnip | Germplasm | 8.39 |
| 101 | ssp. *chinensis* | Pak choi | Germplasm | 2.59 |
| 102 | ssp. *chinensis* | Pak choi | DH line | 2.21 |
| 103 | ssp. *chinensis* | Pak choi | DH line | 2.4 |
| 104 | ssp. *chinensis* | Pak choi | Inbred line | 2.74 |
| 105 | ssp. *chinensis* | Pak choi | DH line | 2.62 |
| 106 | ssp. *chinensis* | Pak choi | DH line | 2.97 |
| 107 | ssp. *chinensis* | Pak choi | Inbred line | 1.59 |
| 108 | ssp. *chinensis* | Pak choi | DH line | 6.66 |
| 109 | ssp. *chinensis* | Pak choi | Inbred line | 1.52 |
| 110 | ssp. *chinensis* | Pak choi | DH line | 2.21 |
| 111 | ssp. *chinensis* | Pak choi | DH line | 2.59 |
| 112 | ssp. *chinensis* | Pak choi | DH line | 2.46 |
| 113 | ssp. *chinensis* | Pak choi | DH line | 2.44 |
| 114 | ssp. *chinensis* | Pak choi | DH line | 2.33 |
| 115 | ssp. *chinensis* | Pak choi | DH line | 4.02 |
| 116 | ssp. *chinensis* | Pak choi | DH line | 2.37 |
| 117 | ssp. *chinensis* | Pak choi | DH line | 2.76 |
| 118 | ssp. *chinensis* | Pak choi | DH line | 2.65 |
| 119 | ssp. *chinensis* | Pak choi | Inbred line | 2.59 |
| 120 | ssp. *chinensis* | Pak choi | DH line | 1.96 |
| 121 | ssp. *chinensis* | Pak choi | DH line | 2.54 |
| 122 | ssp. *chinensis* | Pak choi | DH line | 2.45 |
| 123 | ssp. *chinensis* | Pak choi | DH line | 2.15 |
| 124 | ssp. *chinensis* | Pak choi | DH line | 2.27 |
| 125 | ssp. *chinensis* | Pak choi | DH line | 2.76 |
| 126 | ssp. *narinosa* | Wutacai | Germplasm | 1.76 |
| 127 | ssp. *narinosa* | Wutacai | DH line | 2.75 |
| 128 | ssp. *narinosa* | Wutacai | Germplasm | 1.93 |
| 129 | ssp. *narinosa* | Wutacai | DH line | 2.57 |
| 130 | ssp. *narinosa* | Wutacai | DH line | 2.31 |
| 131 | ssp. *narinosa* | Wutacai | DH line | 5.34 |
| 132 | ssp. *narinosa* | Wutacai | DH line | 2.28 |
| 133 | ssp. *parachinensis* | Caixin | DH line | 2.69 |
| 134 | ssp. *parachinensis* | Caixin | Germplasm | 3.9 |
| 135 | ssp. *parachinensis* | Caixin | Germplasm | 5.76 |
| 136 | ssp. *parachinensis* | Caixin | Germplasm | 7.47 |
| 137 | ssp. *parachinensis* | Caixin | Germplasm | 4.82 |
| 138 | ssp. *parachinensis* | Caixin | Germplasm | 4.6 |
| 139 | ssp. *parachinensis* | Caixin | Germplasm | 4.73 |
| 140 | ssp. *parachinensis* | Caixin | Germplasm | 14.47 |
| 141 | ssp. *parachinensis* | Caixin | Germplasm | 7.69 |
| 142 | ssp. *parachinensis* | Caixin | Germplasm | 7.02 |
| 143 | ssp. *parachinensis* | Caixin | Germplasm | 10.35 |
| 144 | ssp. *parachinensis* | Caixin | Germplasm | 9.5 |
| 145 | ssp. *parachinensis* | Caixin | Germplasm | 5.62 |
| 146 | ssp. *parachinensis* | Caixin | Germplasm | 8.82 |
| 147 | ssp. *parachinensis* | Caixin | Germplasm | 7.69 |
| 148 | ssp. *parachinensis* | Caixin | Germplasm | 11.09 |
| 149 | ssp. *parachinensis* | Caixin | Germplasm | 5.43 |
| 150 | ssp. *parachinensis* | Caixin | Germplasm | 4.73 |
| 151 | ssp. *parachinensis* | Caixin | Germplasm | 5.83 |
| 152 | ssp. *parachinensis* | Caixin | Germplasm | 9.41 |
| 153 | ssp. *parachinensis* | Caixin | Germplasm | 6.98 |
| 154 | ssp. *parachinensis* | Caixin | Germplasm | 6.55 |
| 155 | ssp. *parachinensis* | Caixin | Germplasm | 7.93 |
| 156 | ssp. *parachinensis* | Caixin | Germplasm | 7.53 |
| 157 | ssp. *parachinensis* | Caixin | Germplasm | 4.95 |
| 158 | ssp. *parachinensis* | Caixin | Germplasm | 5.69 |
| 159 | ssp. *parachinensis* | Caixin | Germplasm | 4.81 |
| 160 | ssp. *parachinensis* | Caixin | Germplasm | 7.51 |
| 161 | ssp. *parachinensis* | Caixin | Germplasm | 8.94 |
| 162 | ssp. *parachinensis* | Caixin | Germplasm | 4.43 |
| 163 | ssp. *chinensis*  *var. purpurea* Bailey | Zicaitai | Germplasm | 4.25 |
| 164 | ssp. *chinensis*  *var. purpurea* Bailey | Zicaitai | Germplasm | 3.75 |
| 165 | ssp. *chinensis*  *var. purpurea* Bailey | Zicaitai | Germplasm | 6.91 |
| 166 | ssp. *chinensis*  *var. purpurea* Bailey | Zicaitai | Germplasm | 7.33 |
| 167 | ssp. *chinensis*  *var. purpurea* Bailey | Zicaitai | Germplasm | 5.59 |
| 168 | ssp. *chinensis*  *var. purpurea* Bailey | Zicaitai | Germplasm | 6.5 |
| 169 | ssp. *chinensis*  *var. purpurea* Bailey | Zicaitai | Germplasm | 5.61 |
| 170 | ssp. *chinensis*  *var. purpurea* Bailey | Zicaitai | Germplasm | 6.81 |
| 171 | ssp. *chinensis*  *var. purpurea* Bailey | Zicaitai | Germplasm | 8.03 |
| 172 | ssp. *chinensis*  *var. purpurea* Bailey | Zicaitai | Germplasm | 7.44 |
| 173 | ssp. *chinensis*  *var. purpurea* Bailey | Zicaitai | Germplasm | 4.44 |
| 174 | ssp. *chinensis*  *var. purpurea* Bailey | Zicaitai | Germplasm | 4.32 |
| 175 | ssp. *chinensis*  *var. purpurea* Bailey | Zicaitai | Germplasm | 4.75 |
| 176 | ssp. *chinensis*  *var. tai-tsai Lin* | Taicai | DH line | 10.52 |
| 177 | ssp. *chinensis*  *var. tai-tsai Lin* | Taicai | DH line | 2.6 |
| 178 | ssp. *chinensis*  *var. tai-tsai Lin* | Taicai | DH line | 2.63 |
| 179 | ssp. *chinensis*  *var. tai-tsai Lin* | Taicai | DH line | 2.17 |
| 180 | ssp. *perviridis* | Komatsuna | DH line | 4.95 |
| 181 | ssp. *perviridis* | Komatsuna | DH line | 2.47 |
| 182 | ssp. *nipposinica* | Mizuna | Inbred line | 2.54 |
| 183 | ssp. *nipposinica* | Mizuna | Inbred line | 2.34 |
| 184 | ssp. *broccoletto* | Edible Flower | DH line | 2.69 |
| 185 | ssp. *tricolaris* | Yellow Sarson | Inbred line | 6.39 |
| 186 | ssp. *oleifera* | Oil seeds | Germplasm | 19.08 |
| 187 | ssp. *oleifera* | Oil seeds | Germplasm | 4.58 |
| 188 | ssp. *oleifera* | Oil seeds | Germplasm | 6.36 |
| 189 | ssp. *oleifera* | Oil seeds | Germplasm | 8.23 |
| 190 | ssp. *oleifera* | Oil seeds | Germplasm | 5.18 |
| 191 | ssp. *oleifera* | Oil seeds | Germplasm | 5.57 |
| 192 | ssp. *oleifera* | Oil seeds | Germplasm | 4.73 |
| 193 | ssp. *oleifera* | Oil seeds | Germplasm | 5.64 |
| 194 | ssp. *oleifera* | Oil seeds | Germplasm | 4.67 |
| 195 | ssp. *oleifera* | Oil seeds | Germplasm | 12.05 |
| 196 | ssp. *oleifera* | Oil seeds | Germplasm | 8.83 |
| 197 | ssp. *oleifera* | Oil seeds | Germplasm | 12.22 |
| 198 | ssp. *oleifera* | Rapid cycling | DH line | 4.75 |
| 199 | ssp. *oleifera* | Rapid cycling | DH line | 2.38 |

**DH: double haploid.*

**Supplementary Table 2**. The resequencing and material information of 119 B. oleracea accessions.

| **Index** | **Name** | **English name**  **morphotype** | **Type** | **Resequencing**  **data (Gb)** |
| --- | --- | --- | --- | --- |
| 1 | var. *capitata* | Cabbage | Inbred line | 6.87 |
| 2 | var. *capitata* | Cabbage | Inbred line | 7.19 |
| 3 | var. *capitata* | Cabbage | Inbred line | 7.05 |
| 4 | var. *capitata* | Cabbage | Inbred line | 7.16 |
| 5 | var. *capitata* | Cabbage | Inbred line | 6.18 |
| 6 | var. *capitata* | Cabbage | Inbred line | 7.06 |
| 7 | var. *capitata* | Cabbage | Inbred line | 6.95 |
| 8 | var. *capitata* | Cabbage | Inbred line | 6.96 |
| 9 | var. *capitata* | Cabbage | Inbred line | 7.04 |
| 10 | var. *capitata* | Cabbage | Germplasm | 3.77 |
| 11 | var. *capitata* | Cabbage | Germplasm | 4.03 |
| 12 | var. *capitata* | Cabbage | Germplasm | 8.72 |
| 13 | var. *capitata* | Cabbage | Germplasm | 3.73 |
| 14 | var. *capitata* | Cabbage | Germplasm | 5.26 |
| 15 | var. *capitata* | Cabbage | Germplasm | 5.49 |
| 16 | var. *capitata* | Cabbage | Germplasm | 4.3 |
| 17 | var. *capitata* | Cabbage | Germplasm | 7.63 |
| 18 | var. *capitata* | Cabbage | Germplasm | 4.23 |
| 19 | var. *capitata* | Cabbage | Germplasm | 6.26 |
| 20 | var. *capitata* | Cabbage | Germplasm | 4.41 |
| 21 | var. *capitata* | Cabbage | Germplasm | 4.24 |
| 22 | var. *capitata* | Cabbage | Germplasm | 5.17 |
| 23 | var. *capitata* | Cabbage | Germplasm | 5.11 |
| 24 | var. *capitata* | Cabbage | Germplasm | 1.95 |
| 25 | var. *capitata* | Cabbage | Germplasm | 2.99 |
| 26 | var. *capitata* | Cabbage | Germplasm | 3.95 |
| 27 | var. *capitata* | Cabbage | Germplasm | 4 |
| 28 | var. *capitata* | Cabbage | Germplasm | 1.92 |
| 29 | var. *capitata* | Cabbage | Inbred line | 6.39 |
| 30 | var. *capitata* | Pointed Cabbage | Inbred line | 7.15 |
| 31 | var. *capitata* | Pointed Cabbage | Inbred line | 8.29 |
| 32 | var. *capitata* | Pointed Cabbage | Inbred line | 7.01 |
| 33 | var. *capitata* | White Cabbage | Inbred line | 7.37 |
| 34 | var. *capitata* | White Cabbage | Inbred line | 7.35 |
| 35 | var. *capitata* | White Cabbage | Inbred line | 7.36 |
| 36 | var. *capitata* | White Cabbage | Inbred line | 7.71 |
| 37 | var. *capitata* | White Cabbage | Inbred line | 7.41 |
| 38 | var. *capitata* | White Cabbage | Inbred line | 6.77 |
| 39 | var. *capitata* | White Cabbage | Inbred line | 7.28 |
| 40 | var. *capitata* | White Cabbage | Inbred line | 7.23 |
| 41 | var. *capitata* | White Cabbage | Inbred line | 7.35 |
| 42 | var. *capitata* | White Cabbage | Inbred line | 5.85 |
| 43 | var. *capitata* | White Cabbage | Inbred line | 5.68 |
| 44 | var. *capitata* | Cabbage | Germplasm | 5.23 |
| 45 | var. *capitata* | Cabbage | Germplasm | 6.04 |
| 46 | var. *gongylodes* | Kohlrabi | Inbred line | 6.91 |
| 47 | var. *gongylodes* | Kohlrabi | Inbred line | 7.56 |
| 48 | var. *gongylodes* | Kohlrabi | Inbred line | 7.21 |
| 49 | var. *gongylodes* | Kohlrabi | Inbred line | 7.49 |
| 50 | var. *gongylodes* | Kohlrabi | Inbred line | 7.48 |
| 51 | var. *gongylodes* | Kohlrabi | Inbred line | 5.14 |
| 52 | var. *gongylodes* | Kohlrabi | Genebank | 6.94 |
| 53 | var. *gongylodes* | Kohlrabi | Genebank | 6.91 |
| 54 | var. *gongylodes* | Kohlrabi | Inbred line | 7.52 |
| 55 | var. *gongylodes* | Kohlrabi | Inbred line | 7.65 |
| 56 | var. *gongylodes* | Kohlrabi | Inbred line | 7.41 |
| 57 | var. *gongylodes* | Kohlrabi | Inbred line | 7.74 |
| 58 | var. *gongylodes* | Kohlrabi | Inbred line | 7.59 |
| 59 | var. *gongylodes* | Kohlrabi | Inbred line | 7.65 |
| 60 | var. *gongylodes* | Kohlrabi | Inbred line | 7.25 |
| 61 | var. *gongylodes* | Kohlrabi | Germplasm | 4.56 |
| 62 | var. *gongylodes* | Kohlrabi | Germplasm | 6.56 |
| 63 | var. *gongylodes* | Kohlrabi | Germplasm | 4.66 |
| 64 | var. *gongylodes* | Kohlrabi | Germplasm | 4.75 |
| 65 | var. *botrytis* | Cauliflower | Inbred line | 7.59 |
| 66 | var. *botrytis* | Cauliflower | Inbred line | 7.63 |
| 67 | var. *botrytis* | Cauliflower | Inbred line | 7.2 |
| 68 | var. *botrytis* | Cauliflower | Inbred line | 7.38 |
| 69 | var. *botrytis* | Cauliflower | Inbred line | 7.24 |
| 70 | var. *botrytis* | Cauliflower | Inbred line | 7.13 |
| 71 | var. *botrytis* | Cauliflower | Inbred line | 7.13 |
| 72 | var. *botrytis* | Cauliflower | Inbred line | 7.31 |
| 73 | var. *botrytis* | Cauliflower | Inbred line | 7.21 |
| 74 | var. *botrytis* | Cauliflower | Inbred line | 6.84 |
| 75 | var. *botrytis* | Cauliflower | Inbred line | 6.86 |
| 76 | var. *botrytis* | Cauliflower | Inbred line | 6.55 |
| 77 | var. *botrytis* | Cauliflower | Inbred line | 7.46 |
| 78 | var. *botrytis* | Cauliflower | Inbred line | 7.54 |
| 79 | var. *botrytis* | Cauliflower | Inbred line | 6.86 |
| 80 | var. *botrytis* | Cauliflower | Germplasm | 4.8 |
| 81 | var. *botrytis* | Cauliflower | Germplasm | 5.03 |
| 82 | var. *botrytis* | Cauliflower | Germplasm | 4.64 |
| 83 | var. *botrytis* | Cauliflower | Germplasm | 4.07 |
| 84 | var. *botrytis* | Cauliflower | Germplasm | 5.32 |
| 85 | var. *italica* | Broccoli | Inbred line | 7.53 |
| 86 | var. *italica* | Broccoli | Inbred line | 7.56 |
| 87 | var. *italica* | Broccoli | Inbred line | 7.23 |
| 88 | var. *italica* | Broccoli | Inbred line | 6.32 |
| 89 | var. *italica* | Broccoli | Inbred line | 6.84 |
| 90 | var. *italica* | Broccoli | Inbred line | 6.92 |
| 91 | var. *italica* | Broccoli | Inbred line | 6.85 |
| 92 | var. *italica* | Broccoli | Inbred line | 7.01 |
| 93 | var. *italica* | Broccoli | Inbred line | 6.91 |
| 94 | var. *italica* | Broccoli | Inbred line | 7.89 |
| 95 | var. *italica* | Broccoli | Genebank | 6.67 |
| 96 | var. *italica* | Broccoli | Genebank | 6.63 |
| 97 | var. *italica* | Broccoli | Inbred line | 6.91 |
| 98 | var. *italica* | Broccoli | Inbred line | 6.91 |
| 99 | var. *italica* | Broccoli | Inbred line | 6.97 |
| 100 | var. *italica* | Broccoli | Inbred line | 6.99 |
| 101 | var. *italica* | Broccoli | Inbred line | 7.17 |
| 102 | var. *italica* | Broccoli | Germplasm | 4.18 |
| 103 | var. *italica* | Broccoli | Germplasm | 4.58 |
| 104 | var. *italica* | Broccoli | Germplasm | 4.13 |
| 105 | var. *italica* | Broccoli | Germplasm | 3.71 |
| 106 | var. *italica* | Broccoli | Germplasm | 4.45 |
| 107 | var. *italica* | Broccoli | Germplasm | 4.67 |
| 108 | var. *alboglabra* | Chinese kale | Germplasm | 5.26 |
| 109 | var. *alboglabra* | Chinese kale | Germplasm | 5.32 |
| 110 | var. *alboglabra* | Chinese kale | Germplasm | 7.28 |
| 111 | var. *alboglabra* | Chinese kale | Germplasm | 3.11 |
| 112 | var. *gemmifera* | Brussels sprouts | Germplasm | 4.64 |
| 113 | var. *gemmifera* | Brussels sprouts | Germplasm | 5.36 |
| 114 | var. *acephala* | Kale | Germplasm | 3.92 |
| 115 | var. *acephala* | Kale | Germplasm | 6.7 |
| 116 | var. *sabellica* | Curly Kale | Inbred line | 7.41 |
| 117 | var. *sabellica* | Curly Kale | Inbred line | 6.85 |
| 118 | wild type | Wild | Genebank | 6.81 |
| 119 | wild type | Wild | Genebank | 6.8 |

**Supplementary Table 3**. The number of polymorphic variants in each morphotype group of *B. rapa*.

| **Morphotype** | **#Accessions** | **#Polymorphic**  **SNP loci** | | **#Polymorphic**  **InDel loci** | |
| --- | --- | --- | --- | --- | --- |
|  |  | **Total** | **Functional** | **Total** | **Functional** |
| Chinese cabbages | 46 | 2,136,036 | 157,701 | 277,397 | 14,682 |
| turnip | 54 | 2,229,198 | 160,604 | 300,959 | 15,830 |
| pak choi | 25 | 2,127,552 | 156,849 | 283,543 | 15,072 |
| caixin | 30 | 2,072,862 | 151,704 | 282,052 | 15,016 |
| zicaitai | 13 | 1,647,004 | 124,737 | 231,185 | 12,419 |
| oil seeds | 14 | 2,124,240 | 154,615 | 286,118 | 15,134 |
| taicai | 4 | 1,268,825 | 106,904 | 167,056 | 9,800 |
| wutacai | 7 | 1,542,161 | 122,803 | 206,966 | 11,309 |
| edible flower | 1 | 105,806 | 4,350 | 5,154 | 264 |
| yellow sarson | 1 | 87,287 | 3,994 | 4,108 | 251 |
| komatsuna | 2 | 867,932 | 70,892 | 106,294 | 6,283 |
| mizuna | 2 | 535,754 | 51,071 | 67,082 | 4,732 |

**Supplementary Table 4**. The number of polymorphic variants in each morphotype group of *B. oleracea*.

| **Morphotype** | **#Accessions** | **#Polymorphic**  **SNP loci** | | **#Polymorphic**  **InDel loci** | |
| --- | --- | --- | --- | --- | --- |
|  |  | **Total** | **Functional** | **Total** | **Functional** |
| cabbage | 45 | 3,646,355 | 147,905 | 385,583 | 14,567 |
| kohlrabi | 19 | 3,536,173 | 143,582 | 379,794 | 14,313 |
| cauliflower | 20 | 3,110,352 | 129,325 | 349,343 | 13,341 |
| broccoli | 23 | 3,063,408 | 125,922 | 349,670 | 13,249 |
| Chinese kale | 4 | 1,464,501 | 62,137 | 163,380 | 6,361 |
| kale | 2 | 1,352,654 | 59,404 | 135,771 | 5,583 |
| Brussels sprouts | 2 | 1,405,089 | 62,631 | 139,032 | 5,803 |
| curly kale | 2 | 1,288,572 | 54,910 | 123,789 | 4,846 |
| wild | 2 | 2,071,311 | 89,587 | 198,699 | 7,830 |

**Supplementary Table 5**. Verification polymorphic state of five SNPs in 281 *B.oleracea* accessions.

| **Index** | **Chromosome** | **Position** | **Genotype*** | **%reference allele** | |
| --- | --- | --- | --- | --- | --- |
|  |  |  |  | **Studied set** | **Validate set** |
| 1 | C01 | 19359588 | A/C | 0.473913043 | 0.353790614 |
| 2 | C03 | 55614166 | G/C | 0.710084034 | 0.739669421 |
| 3 | C08 | 39086188 | A/C | 0.469565217 | 0.74137931 |
| 4 | C03 | 21380638 | A/G | 0.487288136 | 0.545627376 |
| 5 | C07 | 44665963 | T/G | 0.621621622 | 0.242647059 |

**: reference allele/derived allele*
